# Opioid and Methadone Use for Infants With Surgically Treated Necrotizing Enterocolitis

**DOI:** 10.1001/jamanetworkopen.2023.18910

**Published:** 2023-06-22

**Authors:** Olivia A. Keane, Abigail K. Zamora, Shadassa Ourshalimian, Elaa M. Mahdi, Ashley Y. Song, Eugene Kim, Ashwini Lakshmanan, Eugene S. Kim, Lorraine I. Kelley-Quon

**Affiliations:** 1 Division of Pediatric Surgery, Children’s Hospital Los Angeles, Los Angeles, California; 2 Department of Surgery, Keck School of Medicine, University of Southern California, Los Angeles; 3 Department of Mental Health, Johns Hopkins Bloomberg School of Public Health, Baltimore, Maryland; 4 Department of Anesthesia, Children’s Hospital Los Angeles, Los Angeles, California; 5 Division of Neonatology, Children’s Hospital Los Angeles, Los Angeles, California; 6 Department of Population and Public Health Sciences, Keck School of Medicine, University of Southern California, Los Angeles; 7 Department of Surgery, Cedars-Sinai Health System, Los Angeles, California

## Abstract

**Importance:**

Necrotizing enterocolitis (NEC) requiring surgical intervention is the most common reason for surgical procedures in preterm neonates. Opioids are used to manage postoperative pain, with some infants requiring methadone to treat physiologic opioid dependence or wean from nonmethadone opioid treatment during recovery.

**Objective:**

To describe postoperative opioid use and methadone treatment for infants with surgically treated NEC and evaluate postoperative outcomes.

**Design, Setting, and Participants:**

A cohort study of infants with surgically treated NEC admitted from January 1, 2013, to December 31, 2022, to 48 Children’s Hospital Association hospitals contributing data to the Pediatric Health Information System (PHIS) was performed. Infants who received methadone preoperatively, were aged 14 days or less at the time of the operation, had a congenital heart disease–related operation, or died within 90 days of the operation were excluded. Mixed-effects multivariable logistic regression was used to evaluate thresholds for duration of opioid use after the operation associated with methadone treatment and clinical outcomes associated with methadone use were enumerated.

**Exposure:**

Postoperative administration of nonmethadone opioids.

**Main Outcomes and Measures:**

Methadone use and postoperative length of stay, ventilator days, and total parenteral nutrition (TPN) days.

**Results:**

Of the 2037 infants with surgically treated NEC identified, the median birth weight was 920 (IQR, 700.0-1479.5) g; 1204 were male (59.1%), 911 were White (44.7%), and 343 were Hispanic (16.8%). Infants received nonmethadone opioids for a median of 15 (IQR, 6-30) days after the operation and 231 received methadone (11.3%). The median first day of methadone use was postoperative day 18 (IQR, days 9-64) and continued for 28 days (IQR, 14-73). Compared with infants who received nonmethadone opioids for 1 to 5 days, infants receiving 16 to 21 days of opioids were most likely to receive methadone treatment (odds ratio, 11.45; 95% CI, 6.31-20.77). Methadone use was associated with 21.41 (95% CI, 10.81-32.02) more days of postoperative length of stay, 10.80 (95% CI, 3.63-17.98) more ventilator days, and 16.21 (95% CI, 6.34-26.10) more TPN days.

**Conclusions and Relevance:**

In this cohort study of infants with surgically treated NEC, prolonged use of nonmethadone opioids after the operation was associated with an increased likelihood of methadone treatment and increased postoperative length of stay, ventilation, and TPN use. Optimizing postoperative pain management for infants requiring an operation may decrease the need for methadone treatment and improve health care use.

## Key Points

**Question:**

Is nonmethadone opioid treatment after surgical procedures associated with methadone use in infants with surgically treated necrotizing enterocolitis?

**Findings:**

In this cohort study of 2037 infants with surgical necrotizing enterocolitis, 11% of infants required methadone treatment after surgical procedures, with overall use significantly varying by hospital. As the cumulative days an infant received nonmethadone opioids increased, the likelihood of methadone treatment increased.

**Meaning:**

By identifying infants with surgical necrotizing enterocolitis at the highest risk of methadone use, these findings suggest an opportunity to expand neonatal opioid stewardship efforts to improve patient outcomes and health care use.

## Teaser

This cohort study evaluates postoperative opioid use and methadone use in infants with surgically treated necrotizing enterocolitis, identifies hospital-level variations in methadone use, and examines postoperative outcomes for infants receiving methadone.

## Introduction

Opioids are a cornerstone of pain management in neonates.^1^ However, long-term complications of prolonged opioid use in neonates include impaired cerebellar growth^2^ and increased rates of intraventricular hemorrhage, periventricular leukomalacia, and death.^3^ Furthermore, prolonged opioid use may require several weeks of methadone treatment to prevent or treat symptoms of opioid dependence or wean from opioids.^4,5,6^ Both extended opioid use and the need for methadone treatment in neonates during hospitalization contribute to prolonged hospitalization and increased costs.^7^

Most current literature on prolonged infant opioid use^8,9,10^ focuses on infants with neonatal abstinence syndrome born to opioid-dependent mothers, excluding infants with prolonged iatrogenic opioid use secondary to physician-prescribed opioids.^11^ There are few epidemiologic studies describing prolonged opioid use and dependence in infants following surgical procedures,^5^ and, to our knowledge, no studies examining methadone use and neonatal outcomes. Surgical interventions in high-risk preterm neonates require comprehensive postoperative pain management strategies, but extended use of opioids resulting in methadone use may be associated with substantial increases in health care use and long-term developmental delays.

Necrotizing enterocolitis (NEC) is an acute condition predominantly affecting preterm neonates that necessitates surgical procedures in up to 50% of the patients.^12,13,14^ Our aim was to evaluate postoperative opioid use and methadone use in infants with surgically treated NEC, identify hospital-level variations in methadone use, and examine postoperative outcomes for infants receiving methadone.

## Methods

### Study Design, Participants, and Data Collection Procedures

A retrospective cohort study was conducted using the Pediatric Health Information System (PHIS). The Children’s Hospital Association maintains the PHIS database, which includes clinical and resource use data for inpatient and outpatient encounters for more than 48 US children’s hospitals. The regional distribution of the 48 institutions is South, 17; Northeast, 7; Midwest, 14; and West, 10. All data are deidentified, and data integrity is checked by the PHIS data quality program. The study was approved by the Children’s Hospital Los Angeles Institutional Review Board. Waiver of informed consent was granted due to the use of deidentified data from PHIS. This study followed the Strengthening the Reporting of Observational Studies in Epidemiology (STROBE) reporting guideline.

Infants with a diagnosis of NEC who underwent surgical management between January 1, 2013, and December 31, 2022, were identified using *International Classification of Diseases, 9th Revision, Clinical Modification* (*ICD-9-CM*) and *International Statistical Classification of Diseases and Related Health Problems, 10th Revision, Clinical Modification* (*ICD-10-CM*) codes, which encompassed an array of abdominal surgical procedures that could be performed in the setting of NEC (eg, laparotomy, bowel resection, ostomy, Penrose drain placement) (eTable 1 in Supplement 1).^15,16^ This definition of surgically managed NEC was based on previous definitions in the literature.^15,16^ Infants who underwent an operation at less than or equal to 14 days of life were excluded to ensure the cohort was not capturing infants with spontaneous intestinal perforation instead of surgically managed NEC.^17,18,19^ Additionally, infants who underwent a congenital heart disease surgical procedure at risk for decreased intestinal perfusion or exposed to methadone preoperatively were excluded. Infants who died within 90 days of the first NEC operation were excluded to facilitate evaluation of postoperative outcomes.^20^ Infants with missing pharmacy data were excluded.

### Definition of Opioid and Methadone Use

Postoperative opioid use was defined as the receipt of any nonmethadone opioid medication^21^ following the first NEC-related operation (eTable 2 in Supplement 1). Postoperative methadone treatment was similarly defined. Receipt of nonmethadone opioids and methadone was determined from PHIS pharmacy billing data time stamped for date of use, enabling evaluation of the date of medication administration and duration of use. Dosage strength and frequency of administration are not captured by the PHIS and were not included in the analysis. Total number of days an infant received nonmethadone opioids and methadone in the 3 weeks following the operation was evaluated. Opioid and methadone use was then categorized by 5-day interval groups (ie, 1-5, 6-10, 11-15, and 16-21 days) and linearly as cumulative use within the designated 21-day period. Patients who only received opioids intraoperatively and did not receive opioids at any other point in the first 21 days postoperatively were not included in the final regression models.

### Definition of Outcomes

The primary outcome of interest was methadone use following a course of postoperative nonmethadone opioid use. A secondary analysis was performed to evaluate the factors that are associated with prolonged opioid use including differences in postoperative length of stay (LOS), postoperative days of ventilator use, and postoperative total parenteral nutrition (TPN) days, between infants with and without methadone use.^22,23,24^

### Definition of First Operation Date and Postoperative Period

The first operation date was identified as the date of the patient’s first abdominal operation (list of surgical procedures included in eTable 1 in Supplement 1). Preoperative use was any medication administration before the first operation date, and the postoperative use period began after the first abdominal operation date. For patients with multiple abdominal operations during hospitalization, the preoperative and postoperative use and outcomes correspond to the first operation date. Final statistical models were adjusted for total number of abdominal operations during hospitalization.

### Patient Characteristics

Patient demographic characteristics were identified through the PHIS and included age at admission, age at first operation date, sex, race and ethnicity, and insurance status. Hospitals participating in the PHIS submit race and ethnicity information according to hospital-specific practices.^25^ These include parent/guardian self-report or assignment at the time of hospital registration. The PHIS categorizes race as Asian, Black, White, and other. The category of other included 2 races or Alaska Native, American Indian, Native Hawaiian, or Pacific Islander due to the low numbers of infants in these categories. Race and ethnicity and insurance status were included in our models as these factors have been shown to influence prescriber decisions to administer opioids in pediatric populations^26,27,28^ and are included in the conceptual framework of Hooten et al^29^ for understanding unintended prolonged opioid use.

Disease severity was measured using version 2 of the pediatric Complex Chronic Conditions (CCC) classification system, representing a medical condition expected to last at least 12 months and involve either several organ systems or 1 organ system severely enough to require specialty pediatric care (eTable 3 in Supplement 1).^30^ The CCC classification system includes 10 categories of diagnoses identified by *ICD-9-CM* codes. To account for confounding secondary to clinical comorbidities, regression models were adjusted for the total number of CCCs present for each patient and grouped as follows for data analysis: 0, 1, 2, 3, and 4 or more CCCs present.

### Statistical Analysis

Continuous variables are described using median (IQR) and were analyzed by Wilcoxon-Mann-Whitney tests. Categorical variables are described by frequency and percentage and were analyzed by χ^2^ tests. Normality for continuous variables was assessed visually by histograms and Q-Q plots. Bivariate analyses were performed to investigate differences in hospital and patient characteristics.

Missing race and ethnicity and birth weight data were handled by multiple imputation using chained equations.^31,32^ This was performed with the assumption that race and ethnicity and birth weight were missing at random. Variables used for imputation included patient and hospital characteristics, such as hospital identification number; hospital region; patient sex, age, and race and ethnicity; and insurance status.

Multivariable linear and logistic regression analysis with mixed effects evaluated factors associated with methadone treatment. Multilevel mixed-effects models were used to control for possible confounding from unmeasured hospital characteristics, such as institutional protocols for methadone use. In each analytic model, patients were nested within hospitals, hospital number was considered a random effect, and patient characteristics were considered as fixed effects.^31,32,33,34,35^ Covariate selection for regression model inclusion was based on clinical assessment, availability from the PHIS, and variables with a significant bivariate association with methadone treatment at 2-sided unpaired *P* < .05. Covariates included sex, age at the time of the operation, birth weight, race and ethnicity, insurance status, hospital region, number of CCCs, total number of abdominal surgical procedures, and year of the operation (eTable 1 in Supplement 1). Additionally, preclinical data suggest there are sex-based^36^ differences in behavior and gene expression^37^ associated with neonatal opioid use. Postoperative LOS was analyzed by comparing results with natural log-transformed outcomes. Results between continuous and log-transformed LOS outcomes were consistent at *P* < .05. Results are presented in nontransformed form for ease of interpretation.

Additionally, a hierarchical generalized linear model with random intercepts was used to evaluate variation in methadone use across institutions by computing the intraclass correlation coefficient. All analyses were conducted with an α = .05 significance threshold. Data were analyzed using SAS software, version 9.4 (SAS Institute Inc) and StataCorp, version 15 (StataCorp LLC).

## Results

### Patient Characteristics

Of the 2037 infants identified (eFigure 1 in Supplement 1), the median birth weight was 920 (IQR, 700.0-1479.5) g, 1204 were male (59.1%), and 833 were female (40.9%) (Table 1). Nearly half of the cohort was White (911 [44.7%]), and 343 (16.8%) were Hispanic. Public insurance was most commonly used, and most infants were hospitalized in the Southern region. The median age at admission was 14 (IQR, 0-33) days with a median age at the time of the operation of 41 (IQR, 25-71) days. Of the total cohort, infants received nonmethadone opioids for a median of 15 (IQR, 6-30) days after the operation. Overall, 231 infants (11.3%) received methadone treatment. Methadone administration was initiated on postoperative day 18 (IQR, 9-64) and continued for 28 (IQR, 14-73) days. Infants exposed to methadone had median postoperative opioid use of 31 (IQR, 18-65) days compared with 13 (IQR, 6-26) days seen in those not receiving methadone (*P* < .001). Postoperative LOS, ventilator use, and TPN dependence were significantly increased for infants receiving methadone.

**Table 1.** Cohort Characteristics and Clinical Outcomes for Infants With Surgical NEC by Postoperative Methadone Use

Variable	Analytic sample (N = 2037)	Methadone use (n = 231)	No methadone (n = 1806)	*P* value
Sex				
Male	1204 (59.1)	139 (60.2)	1065 (59.0)	.73
Female	833 (40.9)	92 (39.8)	741 (41.0)	
Age at admission, median (IQR), d	14 (0-33)	17 (1-35)	14 (0-32)	.04
Age at operation, median (IQR), d	41 (25-71)	43 (28-73)	41 (24-71)	.53
Birth weight, median (IQR), g^a^	920 (700.0-1479.5)	856 (675.0-1360)	930 (700.0-1480)	.11
Ethnicity				
Hispanic or Latino	343 (16.8)	41 (17.7)	302 (16.7)	.92
Not Hispanic or Latino	1511 (74.2)	169 (73.2)	1342 (74.3)	
Unknown	183 (9.0)	21 (9.1)	162 (9)	
Race				
Asian	51 (2.5)	10 (<3.5)	45 (2.5)	.06
Black	644 (31.6)	92 (39.8)	552 (30.6)	
Unknown	117 (5.7)	13 (5.6)	104 (5.8)	
White	911 (44.7)	93 (40.3)	818 (45.3)	
Other^b^	314 (15.4)	27 (11.7)	287 (15.9)	
Insurance				
Private	686 (33.7)	61 (26.4)	625 (34.6)	.04
Public	1309 (64.3)	164 (71.0)	1145 (63.4)	
Other	42 (2.1)	10 (<3.5)	36 (2.0)	
Hospital region				
Midwest	562 (27.6)	51 (22.1)	511 (28.3)	<.001
Northeast	275 (13.5)	10 (<3.5)	267 (14.8)	
South	886 (43.5)	146 (63.2)	740 (41.0)	
West	314 (15.4)	26 (11.3)	288 (15.9)	
Postoperative opioid use, median (IQR), d	15 (6-30)	31 (18-65)	13 (6-26)	<.001
Complex chronic conditions				
0	102 (5.0)	10 (<3.5)	94 (5.2)	<.001
1	305 (15.0)	17 (7.4)	288 (15.9)	
2	619 (30.4)	43 (18.6)	576 (31.9)	
3	533 (26.2)	67 (29.0)	466 (25.8)	
≥4	478 (23.5)	96 (41.6)	382 (21.2)	
No. of NEC-related operations, median (IQR)	1 (1-2)	2 (1-2)	1 (1-2)	<.001
Clinical outcome measures				
Postoperative LOS, median (IQR), d	104 (58-155)	137.5 (92-199)	101 (54-150)	<.001
Postoperative ventilator, median (IQR), d	9 (4-23)	15 (6-37)	8 (4-21.5)	<.001
Postoperative TPN use, median (IQR), d^c^	51 (20-98)	91 (48-142)	46 (18-92)	<.001

Abbreviations: LOS, length of stay; NEC, necrotizing enterocolitis; TPN, total parenteral nutrition.

^a^ Birth weight reported in 1632 infants.

^b^ Other indicates more than 1 race or Alaska Native, American Indian, Native Hawaiian, or Pacific Islander.

^c^ Postoperative TPN use reported in 1968 infants.

### Opioid and Methadone Use

**Figure 1.**
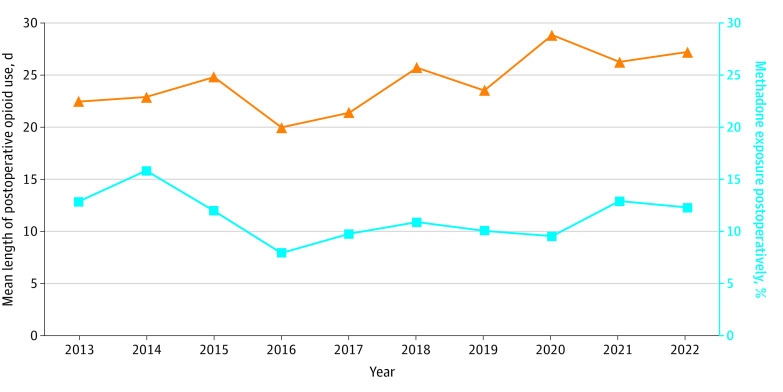
Mean Days of Postoperative Opioid Use and Incidence of Methadone Use

As the mean days of postoperative opioid use increased, the incidence of methadone use likewise increased (Figure 1). The opioid and methadone use curves tracked each other over the study period with a peak around 2020-2021. To account for trend, first-differencing was conducted monthly for the data spanning 2020-2021, as this was the peak of use (eFigure 2 in Supplement 1). The resulting best-fit line had a low to moderately positive Pearson correlation coefficient (*r* = 0.36) and was statistically significant (*P* = .03) (eFigure 3 in Supplement 1). This ultimately suggests that the curves seen in Figure 1 represent a statistically significant difference rather than an observation of data trending.

### Variability of Methadone Use

**Figure 2.**
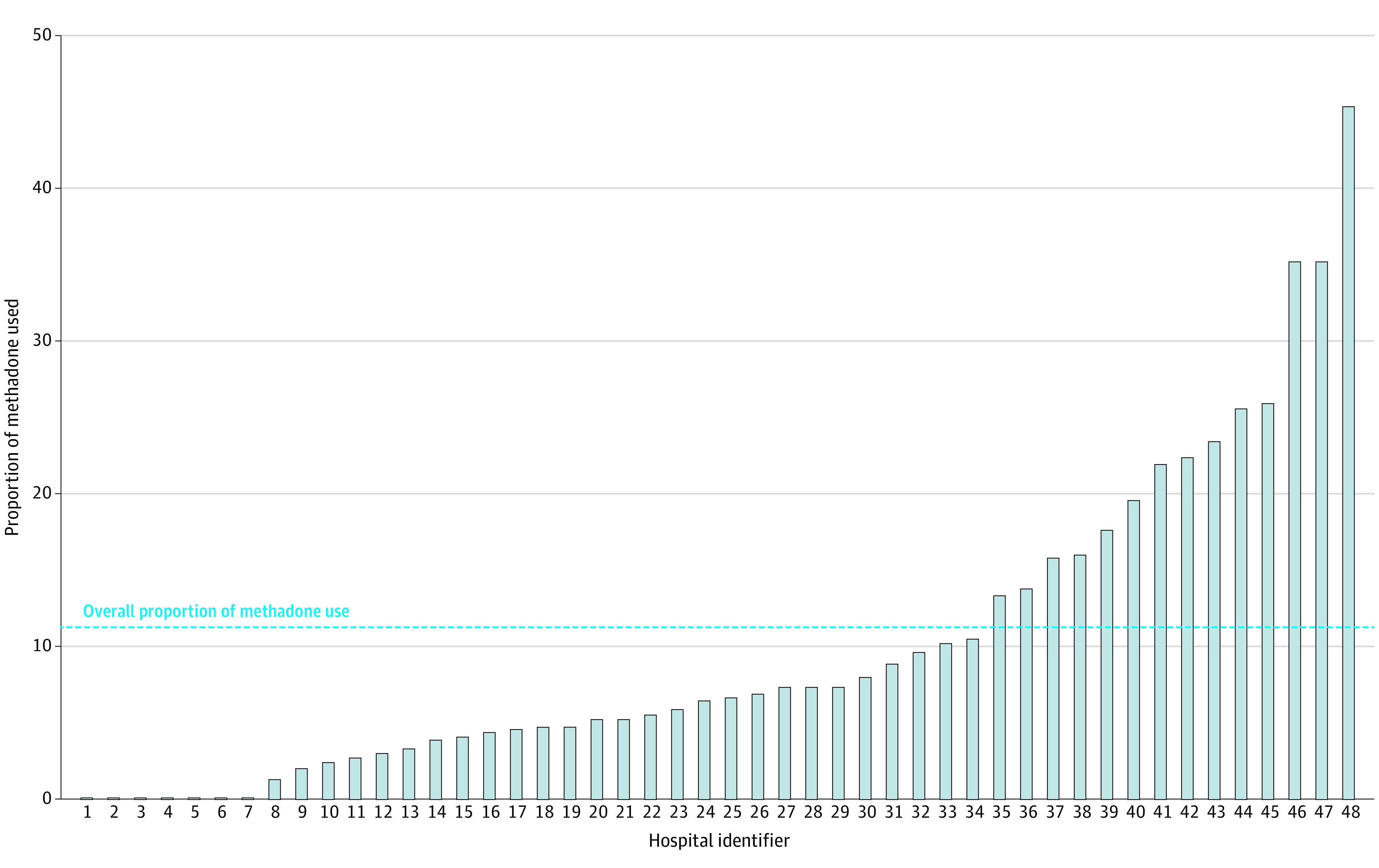
Institutional Variation of Overall Proportion of Methadone Use

There is substantial hospital-level variation in methadone use, with 7 hospitals that do not use methadone (Figure 2). Approximately 25% of the variability in postoperative methadone use for an infant with surgically treated NEC was accounted for by the individual hospital. Therefore, an estimated 75% of the variability remains to be explained by patient or other unknown characteristics. In summary, the unconditional or null model results estimated that the probability of postoperative methadone use at a typical US children’s hospital is approximately 7.0%, with considerable variation in the probability of methadone treatment across hospitals.

### Likelihood of Methadone Use

A multivariable logistic regression analysis of nonmethadone opioid use within the first 21 days postoperatively and associated outcomes was performed (Table 2). As cumulative postoperative opioid days increased, so did the likelihood of requiring methadone after the operation. After adjusting for patient and hospital characteristics, infants receiving 16 to 21 cumulative days of nonmethadone opioids after the surgical procedure had the highest likelihood of receiving methadone compared with infants receiving 1 to 5 days of opioids (odds ratio [OR], 11.45; 95% CI, 6.31-20.77). When opioid use was evaluated as a continuous outcome, each additional day of opioid use was associated with a 15% increase in the likelihood of an infant receiving methadone (OR, 1.15; 95% CI, 1.11-1.18).

**Table 2.** Associations of Postoperative Opioid Use With Methadone Use

Cumulative postoperative opioid use, d	Unadjusted		Adjusted^a^	
	OR (95% CI)	*P* value	OR (95% CI)	*P* value
1-5	1 [Reference]	NA	1 [Reference]	NA
6-10	4.53 (2.57-7.98)	<.001	3.67 (2.03-6.62)	<.001
11-15	6.71 (3.73-12.07)	<.001	4.95 (2.68-9.14)	<.001
16-21	16.62 (9.46-29.20)	<.001	11.45 (6.31-20.77)	<.001

Abbreviations: NA, not applicable; OR, odds ratio.

^a^ Multivariable logistic regression model adjusted for sex, age at the time of the operation, birth weight, race and ethnicity, insurance, year of procedure, postoperative opioid use, number of complex chronic conditions, total number of necrotizing enterocolitis–related operations, and hospital region.

### Methadone Use and Adjusted Outcomes

Adjusting for patient and hospital characteristics, infants receiving methadone postoperatively had 21.41 days longer postoperative LOS (95% CI, 10.81-32.02) and were on the ventilator for 10.80 (95% CI, 3.63-17.98) more days than infants not receiving methadone (Table 3). Additionally, infants receiving methadone postoperatively required 16.21 more days (95% CI, 6.34-26.10 days) of TPN than those not exposed to methadone. Due to the skewed nature of the data, we conducted additional analysis for the natural log-transformed outcomes for postoperative LOS, ventilator days, and TPN use and methadone use (eTable 4 in Supplement 1).

**Table 3.** Postoperative Outcomes Associated With Methadone Use

Variable	Unadjusted		Adjusted^a^	
	Estimate (95% CI)	*P* value	Estimate (95% CI)	*P* value
Length of stay, d	58.89 (46.05-71.74)	<.001	21.41 (10.81-32.02)	<.001
Ventilator use, d	18.30 (9.74-26.84)	<.001	10.80 (3.63-17.98)	<.001
TPN use, d^b^	46.34 (34.51-58.17)	<.001	16.21 (6.34-26.10)	.001

Abbreviation: TPN, total parenteral nutrition.

^a^ Multivariable logistic regression model adjusted for sex, age at the time of the operation, birth weight, race and ethnicity, insurance, year of procedure, postoperative opioid use, number of complex chronic conditions, total number of necrotizing enterocolitis–related surgical procedures, and hospital region.

^b^ Postoperative TPN use reported in 1968 infants.

## Discussion

Infants often receive opioid treatment to manage postoperative pain, and infants who have multiple procedures are likely most in need of opioids and at greater risk of requiring methadone treatment. Our findings highlight a significant temporal association between duration of postoperative nonmethadone opioid use and subsequent methadone treatment, with significant hospital-level variations in methadone use. In addition, overall health care use measured by postoperative LOS, ventilator requirement, and TPN use increased in infants treated with methadone. Prolonged opioid use and subsequent use of methadone adds to the already substantial health care burden of in-hospital costs for surgically managed NEC.^7,16^ These findings suggest a major need for expansion of neonatal opioid stewardship efforts to improve clinical outcomes for infants requiring operations.

Overall, 11.3% of the infants with surgically managed NEC received methadone, and we observed an 11-fold increase in methadone use for infants receiving 16 to 21 days of nonmethadone opioids compared with those receiving less than 6 days of nonmethadone opioids. Notably, all measured metrics of health care use captured in this study were increased for infants receiving methadone. These findings are largely in line with the adverse effect profile of opioids and methadone. Opioids decrease respiratory drive and gut motility, leading to prolonged ventilator days and increased TPN dependence, which can ultimately result in prolonged LOS and likely increased health care use.^22,23,24,38,39,40,41,42^ Our findings suggest that judicious use of opioids in the postoperative period may reduce the need for methadone use and lead to improved patient outcomes.

Methadone treatment as a weaning strategy is often standardized by hospital. As this study highlights, there is wide variability in methadone use across children’s hospitals in the US, with 25% of the variability in methadone treatment accounted for at the hospital level. These findings are consistent with current data on inpatient opioid prescribing in the US and Canada noting wide institutional variation for critically ill infants and children.^4,21,43^ Seven hospitals in the present study did not use methadone and thus likely use nonmethadone weaning protocols. Alternatives to methadone treatment include buprenorphine, dexmedetomidine, clonidine, and gradual tapering of morphine.^44^ It may be that nonmethadone weaning strategies minimize some of the secondary outcomes measured in this study and warrants future investigation. Ultimately, the significant hospital-level variation observed in this study underscores an opportunity for quality improvement interventions to optimize postoperative opioid use and supports future efforts to develop guidelines for pain management for infants undergoing surgical procedures.

### Limitations

This study has limitations. The PHIS is an administrative database and does not include clinical indications for medication administration, dosing of medication, patient pain scores, or timing of a diagnosis of NEC in relation to the surgical procedure. This limited our ability to confirm that methadone was administered in response to clinical signs of opioid withdrawal or that methadone was given as part of a protocol to prevent symptoms of opioid withdrawal or evaluate the cumulative opioid dosage and use of methadone. As NEC typically occurs in preterm infants and their neurologic maturation and physiologic ability to show signs of withdrawal are likely reduced compared with term infants,^45,46,47^ a clinical diagnosis of withdrawal in a preterm neonate with NEC is challenging. Furthermore, surgical indication and timing related to a diagnosis of NEC is not captured by the PHIS and thus we were unable to evaluate whether one of the abdominal surgical procedures included in our study was performed acutely for NEC treatment or for a complication of NEC, such as stricture resection. Similarly, our conclusions may be somewhat limited because infants requiring intubation often receive opioids while intubated,^24^ and prolonged opioid use and subsequent methadone treatment can also contribute to respiratory depression and prolonged ventilation. In addition, a likely factor associated with decreased opioid use for infants undergoing surgical procedures is the use and timing of multimodal pain management strategies, including nonopioid pain medications, regional anesthetics, and nonpharmacologic interventions (eg, swaddling, skin-to-skin care, and breast milk feedings).^38,48,49^ Future studies evaluating patient pain scores alongside timing and use of multimodal pain management strategies to decrease opioid and methadone use are necessary to expand principles of opioid stewardship to neonates undergoing operations.

## Conclusion

In this cohort study of infants with surgically treated NEC, methadone use occurred in 11.3% and varied widely by hospital. Methadone use was characterized by prolonged postoperative hospitalization, ventilator use, and TPN dependence. Further work is needed to better understand the presence or absence of pain and the diagnosis of withdrawal and tolerance in the neonatal population. Judicious use of opioids and multimodal pain management strategies in the postoperative period may reduce the need for methadone, improve patient outcomes, and decrease health care use for infants requiring surgical procedures.

## Notes

Supplement 1. **eFigure 1.** Flow Diagram Demonstrating Cohort Selection **eFigure 2.** First-Difference by Month for the Peak of Opioid and Methadone Use (2020-2022) **eFigure 3.** Scatter Plot for Detrended Data and Pearson Correlations for Change (∆) in Mean Postoperative Opioid Use (Days) and Change (∆) in Percent Postoperative Receipt of Methadone **eTable 1.** *ICD-9* and *ICD-10* Codes Used for Cohort Selection **eTable 2.** Opioid Medication Included **eTable 3.** Complex Chronic Conditions (CCCs) Category by Methadone Use **eTable 4.** Comparison of Postoperative Methadone Use and Associated Adjusted Outcomes in the Postoperative Period, Natural Log-Transformed Outcomes

Supplement 2.
Data Sharing Statement
