# Supplementary material for: Opioid and Methadone Use for Infants With Surgically Treated Necrotizing Enterocolitis
Source: JAMA Netw Open. 2023 Jun 22;6(6):e2318910. doi: 10.1001/jamanetworkopen.2023.18910 (PMC10288332; doi:10.1001/jamanetworkopen.2023.18910)
Supplement: Supplement 1. — eFigure 1. Flow Diagram Demonstrating Cohort Selection eFigure 2. First-Difference by Month for the Peak of Opioid and Methadone Use (2020-2022) eFigure 3. Scatter Plot for Detrended Data and Pearson Correlations for Change (∆) in Mean Postoperative Opioid Use (Days) and Change (∆) in Percent Postoperative Receipt of Methadone eTable 1. ICD-9 and ICD-10 Codes Used for Cohort Selection eTable 2. Opioid Medication Included eTable 3. Complex Chronic Conditions (CCCs) Category by Methadone Use eTable 4. Comparison of Postoperative Methadone Use and Associated Adjusted Outcomes in the Postoperative Period, Natural Log-Transformed Outcomes [file jamanetwopen-e2318910-s001.pdf]

Supplementary Online Content

Keane OA, Zamora AK, Ourshalimian S, et al. Opioid and methadone use for infants with surgically treated necrotizing enterocolitis. *JAMA Netw Open*. 2023;6(6):e2318910. doi:10.1001/jamanetworkopen.2023.18910

eFigure 1. Flow Diagram Demonstrating Cohort Selection

eFigure 2. First-Difference by Month for the Peak of Opioid and Methadone Use (2020-2022)

eFigure 3. Scatter Plot for Detrended Data and Pearson Correlations for Change (Δ) in Mean Postoperative Opioid Use (Days) and Change (Δ) in Percent Postoperative Receipt of Methadone

eTable 1. *ICD-9* and *ICD-10* Codes Used for Cohort Selection

eTable 2. Opioid Medication Included

eTable 3. Complex Chronic Conditions (CCCs) Category by Methadone Use

eTable 4. Comparison of Postoperative Methadone Use and Associated Adjusted Outcomes in the Postoperative Period, Natural Log-Transformed Outcomes

This supplementary material has been provided by the authors to give readers additional information about their work.

© 2023 Keane OA et al. *JAMA Network Open.*

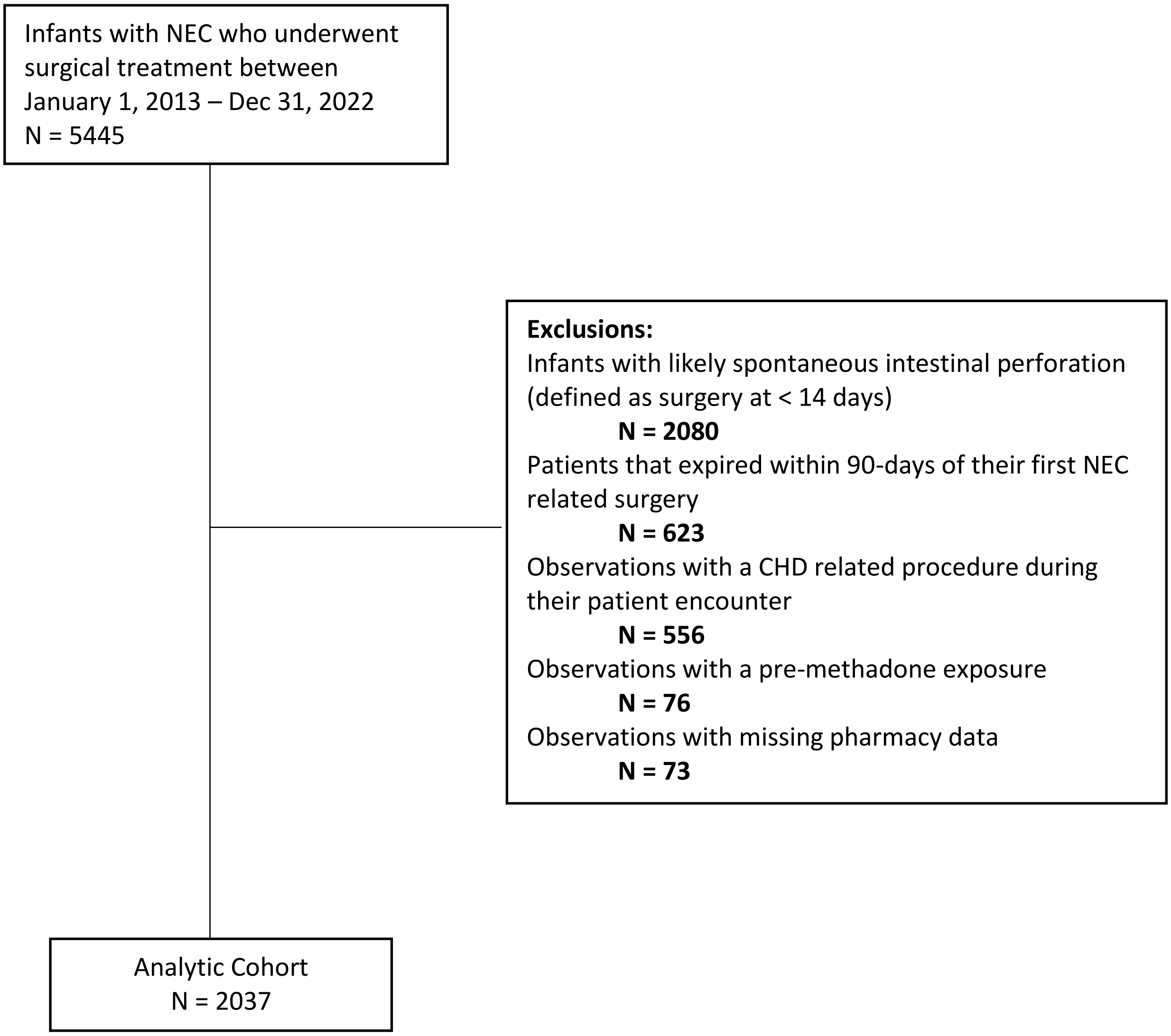

eFigure 1. Flow diagram demonstrating cohort selection

© 2023 Keane OA et al. *JAMA Network Open.*

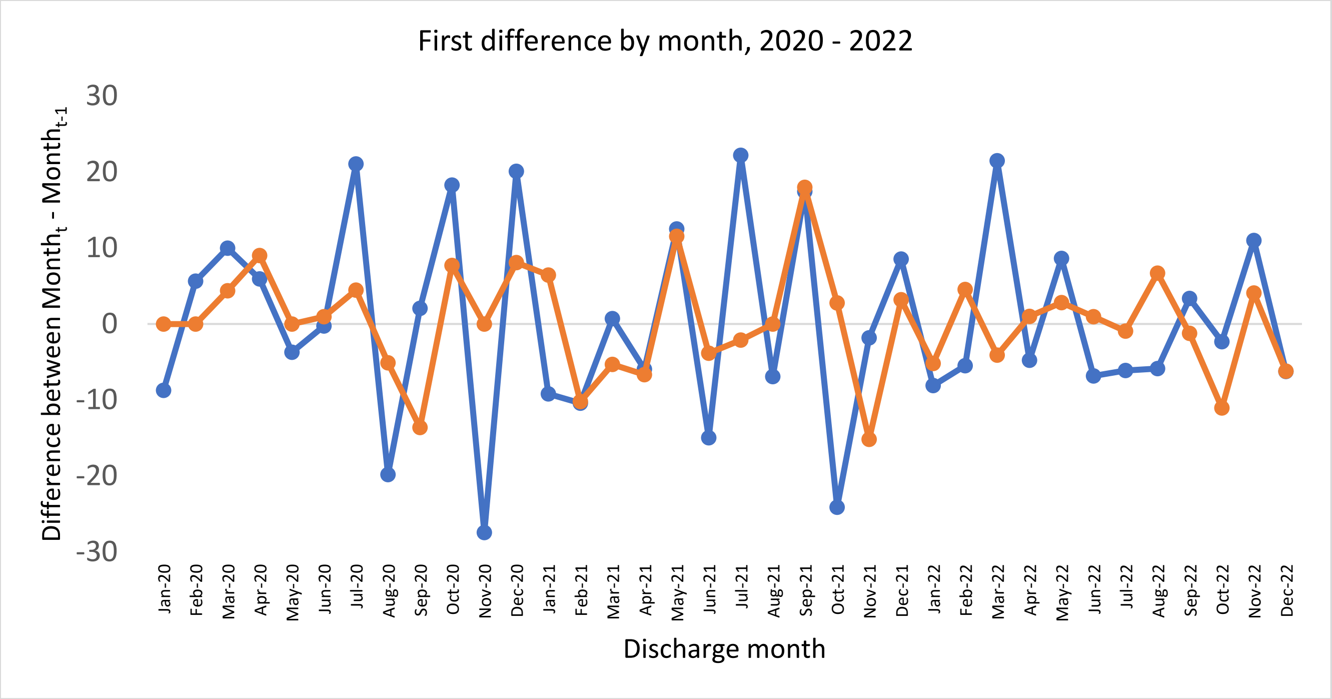

eFigure 2. First-difference by month for the peak of opioid and methadone use (2020-2022)

Blue triangle = mean opioid use (days). Orange square = percent methadone (%).

© 2023 Keane OA et al. *JAMA Network Open.*

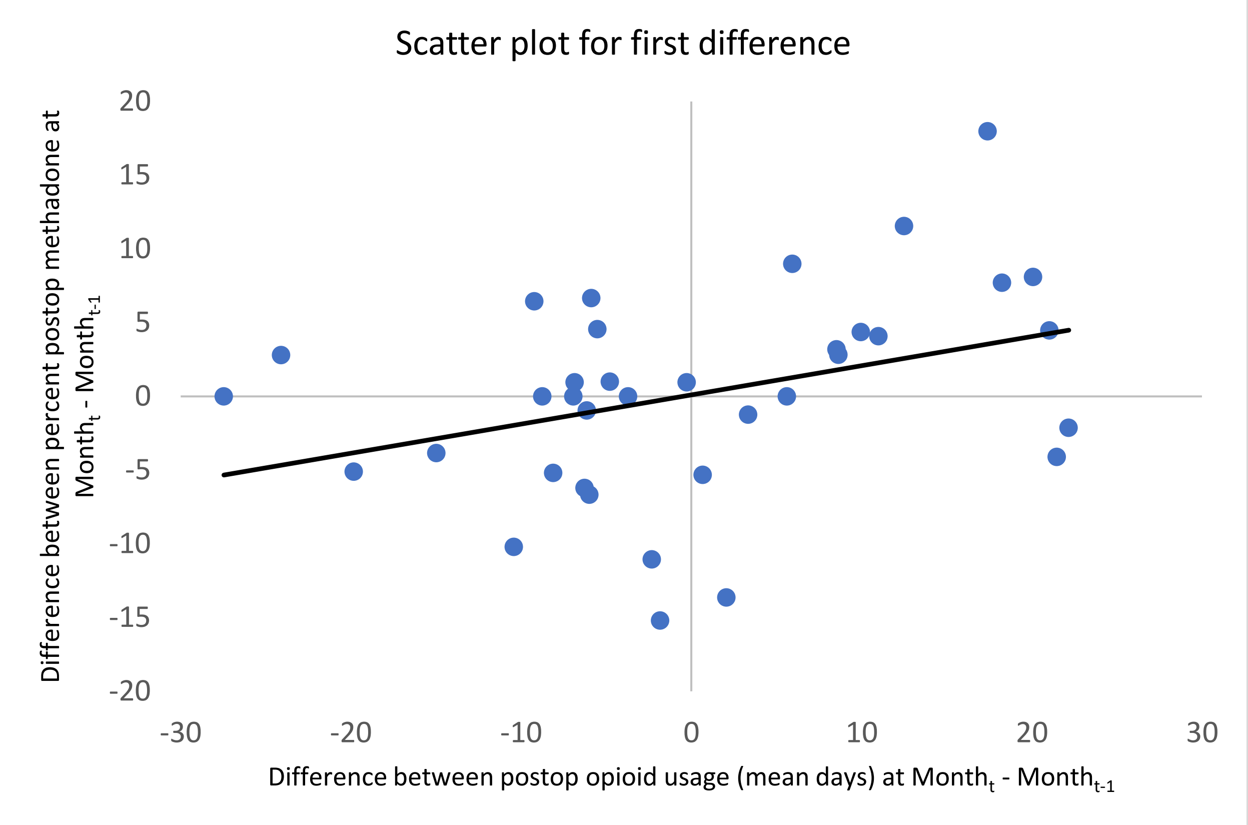

eFigure 3. Scatter plot for detrended data and Pearson correlations for change (Δ) in mean postoperative opioid use (days) and change (Δ) in percent postoperative receipt of methadone. Legend: There was a statistically significant ($p=0.03$) low to moderate positive correlation ($r = 0.36$) for change (Δ) in mean postoperative opioid use (days) and change (Δ) in percent of patients who received postoperative methadone.

© 2023 Keane OA et al. *JAMA Network Open.*

eTable 1. ICD-9 and ICD-10 codes used for cohort selection.

Variable	ICD-9 Code	Variable	ICD-10 Code
**Diagnoses**		**Diagnoses**	
NEC	777.5, 777.50, 777.51 – 777.53	NEC	P77.1, P77.2, P77.3, P77.9, K55.30, K55.31, K55.32, K55.33
**Procedures**		**Procedures**	
Enterotomy	45.00 - 45.03	Excision or Resection of Small Intestine	0DB80ZX, 0DB80ZZ, 0DB84ZX, 0DB84ZZ, 0DT80ZZ, 0DT84ZZ
Diagnostic procedures on small intestine	45.1, 45.15	Excision or Resection of Duodenum	0DB90ZX, 0DB90ZZ, 0DB94ZX, 0DB94ZZ, 0DT90ZZ, 0DT94ZZ
Diagnostic procedures on large intestine	45.2, 45.26	Excision or Resection of Jejunum	0DBA0ZX, 0DBA0ZZ, 0DBA4ZX, 0DBA4ZZ, 0DTA0ZZ, 0DTA4ZZ
Local excision or destruction of lesion or tissue of small intestine	45.3 - 45.34	Excision or Resection of Ileum	0DBB0ZX, 0DBB0ZZ, 0DBB4ZX, 0DBB4ZZ, 0DTB0ZZ, 0DTB4ZZ
Local excision or destruction of lesion or tissue of large intestine	45.4, 45.41, 45.49	Drainage of Peritoneum	0D9W00Z,<br>0D9W0ZX, 0D9W0ZZ, 0D9W40Z, 0D9W4ZX, 0D9W4ZZ
Isolation of intestinal segment	45.5, 45.50	Inspection of Peritoneum	0DJW0ZZ, 0DJW4ZZ
Incision, excision, and anastomosis of intestine	45.5, 45.50, 45.52	Excision of Large Intestine	0DBE0ZX, 0DBE0ZZ, 0DBE4ZX, 0DBE4ZZ, 0DBF0ZX, 0DBF0ZZ, 0DBF4ZX, 0DBF4ZZ, 0DBG0ZX, 0DBG0ZZ, 0DBG4ZX, 0DBG4ZZ, 0DBGFZZ
Other excision of small intestine	45.61 - 45.63	Excision or Resection of Cecum	0DBH0ZX, 0DBH0ZZ, 0DBH4ZX, 0DBH4ZZ, 0DTH0ZZ, 0DTH4ZZ
Open and other partial excision of large intestine	45.7 - 45.79	Excision or Resection of Ascending Colon	0DBK0ZX, 0DBK0ZZ, 0DBK4ZX, 0DBK4ZZ, 0DTK0ZZ, 0DTK4ZZ
Total intra-abdominal colectomy	45.8 - 45.83	Excision or Resection of Transverse Colon	0DBL0ZX, 0DBL0ZZ, 0DBL4ZX, 0DBL4ZZ, 0DBLFZZ, 0DTL0ZZ, 0DTL4ZZ
Intestinal anastomosis	45.9	Excision or Resection of Descending Colon	0DBM0ZX, 0DBM0ZZ, 0DBM4ZX, 0DBM4ZZ, 0DBMFZZ, 0DTM0ZZ, 0DTM4ZZ

© 2023 Keane OA et al. *JAMA Network Open.*

Colostomy	46.13, 46.14	Excision or Resection of Sigmoid Colon	0DBN0ZX, 0DBN0ZZ, 0DBN4ZX, 0DBN4ZZ, 0DBNFZZ, 0DTN0ZZ, 0DTN4ZZ
Ileostomy	46.2 - 46.24		
Other enterostomy	46.3 - 46.39		
Revision of intestinal stoma	46.41 - 46.43		
Closure of intestinal stoma	46.5 - 46.52		
Fixation of intestine	46.6 - 46.64		
Other repair of intestine	46.7 - 46.79		
Laparotomy	54.1 - 54.19		
Diagnostic procedures of abdominal region	54.2, 54.25		
Excision or destruction of peritoneal tissue	54.4		
Lysis of peritoneal adhesions	54.51, 54.59		
Suture of abdominal wall and peritoneum	54.6 - 54.64		
Other operations of abdominal region	54.91, 54.93		

NEC=Necrotizing enterocolitis

© 2023 Keane OA et al. *JAMA Network Open.*

eTable 2. Opioid medication included

**Opioid Medications Examined:**	Alfentanil HCl<br>Fentanyl citrate<br>Hydromorphone HCl<br>Meperidine HCl<br>Oxycodone HCl<br>Remifentanil HCl<br>Sufentanil citrate<br>Nalbuphine HCl<br>Narcotic analgesic combinations

© 2023 Keane OA et al. *JAMA Network Open.*

eTable 3. Complex Chronic Conditions (CCCs) category by methadone use

	Overall<br>N = 2037 (%)	Methadone Use<br>N = 231 (%)	No Methadone<br>N = 1806 (%)
**Complex Chronic Conditions**			
Cardiovascular	581 (28.5)	98 (42.4)	483 (26.7)
Congenital or Genetic Defect	185 (9.1)	30 (13)	155 (8.6)
Gastrointestinal	1554 (76.3)	181 (78.4)	1373 (76)
Malignancy	27 (1.3)	10 (<3.5)	24 (1.3)
Metabolic	323 (15.9)	58 (25.1)	265 (14.7)
Neurologic & Neuromuscular	351 (17.2)	61 (26.4)	290 (16.1)
Premature/Neonatal	1551 (76.1)	187 (81)	1364 (75.5)
Renal & Urologic	394 (19.3)	59 (25.5)	335 (18.5)
Respiratory	307 (15.1)	69 (29.9)	238 (13.2)

© 2023 Keane OA et al. *JAMA Network Open.*

eTable 4. Comparison of postoperative methadone use and associated adjusted outcomes in the postoperative period, natural log-transformed outcomes

	Receipt of Postop Methadone (Y/N)					
	Unadjusted			Adjusted[a]		
	Estimate	p-value	95% CI	Estimate	p-value	95% CI
Postoperative LOS, days	1.74	<.001	(1.55, 1.95)	1.12	.007	(1.03, 1.21)
Postoperative ventilator use, days	2.02	<.001	(1.67, 2.45)	1.27	.005	(1.08, 1.50)
Postoperative TPN, days[b]	2.08	<.001	(1.81, 2.37)	1.17	.002	(1.06, 1.30)

[a] Final model adjusted for sex, age at surgery, birthweight, ethnicity, race, insurance, year of procedure, postop opioid use, number of complex chronic conditions, total number of NEC-related surgeries, and hospital region.

[b]N=1968

© 2023 Keane OA et al. *JAMA Network Open.*
