# Supplementary material for: Opioid and Methadone Use for Infants With Surgically Treated Necrotizing Enterocolitis
Source: JAMA Netw Open. 2023 Jun 22;6(6):e2318910. doi: 10.1001/jamanetworkopen.2023.18910 (PMC10288332; doi:10.1001/jamanetworkopen.2023.18910)
Supplement: Supplement 2. — Data Sharing Statement [file jamanetwopen-e2318910-s002.pdf]

Data Sharing Statement

Keane. Opioid and Methadone Use for Infants With Surgically Treated Necrotizing Enterocolitis. *JAMA Netw Open*. Published June 22, 2023.
doi:10.1001/jamanetworkopen.2023.18910

Data
Data available: Yes
Data types: Deidentified participant data, Data dictionary
How to access data: lkquon@chla.usc.edu
When available: With publication

Supporting Documents
Document types: Statistical/analytic code
How to access documents: lkquon@chla.usc.edu
When available: With publication

Additional Information
Who can access the data: Researchers whose proposed use of the data has been approved
Types of analyses: for those hoping to expand on this area of research
Mechanisms of data availability: after approval of a proposal and with a signed data access agreement
